# Towards Reliable Methodology: Microbiome Analysis of Fresh Frozen vs. Formalin-Fixed Paraffin-Embedded Bladder Tissue Samples: A Feasibility Study

**DOI:** 10.3390/microorganisms12122594

**Published:** 2024-12-15

**Authors:** Dominik Enderlin, Uwe Bieri, Jana Gadient, Yasser Morsy, Michael Scharl, Jan Hendrik Rüschoff, Lukas John Hefermehl, Anna Nikitin, Janine Langenauer, Daniel Stephan Engeler, Beat Förster, Fabian Obrecht, Jonathan Surber, Thomas Paul Scherer, Daniel Eberli, Cédric Poyet

**Affiliations:** 1 Department of Urology, University Hospital of Zurich, University of Zurich, 8091 Zurich, Switzerland; 2 Division of Urology, Department of Surgery, Kantonsspital Baden, 5404 Baden, Switzerland; 3 Department of Gastroenterology and Hepatology, University Hospital of Zurich, University of Zurich, 8091 Zurich, Switzerland; 4 Department of Pathology, and Molecular Pathology, University Hospital of Zurich, University of Zurich, 8091 Zurich, Switzerland; 5 Department of Urology, Cantonal Hospital of St. Gallen, School of Medicine, University of St. Gallen, 9007 St. Gallen, Switzerland; 6 Department of Urology, Kantonsspital Winterthur, 8401 Winterthur, Switzerland

**Keywords:** fresh frozen, formalin-fixed paraffin-embedded, microbiome, bladder cancer, alpha diversity

## Abstract

Studies have shown that the human microbiome influences the response to systemic immunotherapy. However, only scarce data exist on the impact of the urinary microbiome on the response rates of bladder cancer (BC) to local *Bacillus Calmette-Guérin* instillation therapy. We launched the prospective SILENT-EMPIRE study in 2022 to address this question. We report the results of the pilot study of SILENT-EMPIRE, which aimed to compare the microbiome between fresh frozen (FF) and formalin-fixed paraffin-embedded (FFPE) samples in the cancerous tissue and adjacent healthy tissue of BC patients. Our results show that alpha diversity is increased in FF samples compared to FFPE (coverage index *p* = 0.041, core abundance index *p* = 0.008). No significant differences concerning alpha diversity could be detected between cancerous and non-cancerous tissue in the same BC patients. This study demonstrates that microbiome analysis from both FF and FFPE samples is feasible. Implementing this finding could aid in the translation of research findings into clinical practice.

## 1. Introduction

Bladder cancer (BC) remains the 10th most common cancer worldwide, with over 500,000 new cases annually [1]. Around 70% of those cases are diagnosed early as non-muscle-invasive bladder cancer (NMIBC) [2,3]. NMIBC is usually treated with local transurethral resection. Still, there is a high chance of local recurrence of 50% and a distinct risk of progression to a muscle-invasive disease (MIBC) of around 10–20% [3,4,5]. Therefore, adjuvant local therapy with *Bacillus Calmette-Guérin* (BCG) bladder instillations is often performed to minimize the risk of disease recurrence and progression in higher-risk cases. The BCG mechanism is understood to work via an immune system activation [6]. However, a relevant number of patients experience cancer recurrence despite BCG treatment [7]. High-risk features such as increased tumour size, multifocality, the presence of carcinoma in situ (CIS), and lymphovascular invasion are factors that increase the risk of BCG failure, but many are still unknown, and to date, no established predictive biomarkers to anticipate therapeutic response are available [8,9]. Hence, improving the identification of patients who will benefit from BCG and those who will not in the clinical treatment of NMIBC is crucial [7].

In recent years, the urinary tract microbiome has shifted into the focus of oncology research [10]. Several studies have shown differences in the urinary microbiome of BC patients compared to healthy individuals, even though the results so far are inconsistent [11,12,13]. In other cancer types, it has been demonstrated that the human microbiome influences the response to different systemic cancer therapies, including immunotherapy [14,15,16]. Data on how the urinary microbiome influences response to BCG remain scarce.

We have launched the SILENT-EMPIRE study to address this pending issue and evaluate the possible application of the bladder microbiome as a predictive marker for BCG therapy response. The primary objective of this prospective, longitudinal, observational study is to assess differences in the microbiome composition of different sample types, including BC tissue, for possible predictive information concerning responses to BCG therapy [17].

In the clinical routine, formalin-fixed paraffin-embedded (FFPE) samples are generally used for histopathological assessment, whereas fresh frozen (FF) samples are used for intraoperative frozen section examination [18,19]. While both methods have advantages in the context of clinical research, FFPE is often the preferred choice due to its more straightforward production process [20]. This involves immersing the tissue sample in formalin to preserve its structures and embedding it in paraffin wax for easier cutting and handling. On the other hand, FF samples are stored in their natural state without any chemical treatment, allowing for better preservation of biomolecules such as DNA and RNA [20]. However, this method requires specialized equipment and trained personnel, making it less accessible than FFPE. Nevertheless, using FF samples is crucial in certain studies that require the analysis of specific biomolecules.

In prior experiments, it was shown that more modification of the proteomic profile occurred in FFPE samples compared to FF-processed samples [21]. A study conducted by Wang et al. [22] found that DNA extraction, a crucial step in the analysis of the microbiome, was more successful in FF tissue samples compared to FFPE samples.

The impact of sample fixation and storage on the detected microbiome in bladder tissue has received limited attention in the scientific literature. While there is a growing body of research focused on investigating the microbiome of various tissues and organs, there remains a notable gap in our understanding of the potential influence of sample preparation and storage methods on the accuracy and reliability of microbiome data. Therefore, further studies examining these factors and standardizing protocols for sample handling are needed to accurately characterize the microbiome of bladder tissue and its potential role as a biomarker and in disease pathology.

In this pilot study, we aimed to examine the feasibility of assessing the microbiome of bladder tissue samples by comparing two different storage techniques: FF and FFPE. Our primary objective was to determine if there were any notable variations in microbiome biodiversity between the two storage methods and, secondly, to provide more insight into the best practices for handling and storing tissue samples for microbiome analysis, ultimately providing crucial guidance for the design and interpretation of future studies.

## 2. Material and Methods

### 2.1. Sample Acquisition and Analysis

All patients at the University Hospital of Zurich with cystoscopic suspicion for BC were screened according to the inclusion and exclusion criteria in the previously published study protocol [17]. After obtaining informed consent, the samples were collected during the transurethral tumour resection. Tumour tissue and adjacent normal tissue were collected from each patient; of each tissue type, one part was archived by formalin fixation and three parts by fresh freezing. According to the protocol, the non-cancerous tissue was collected at a 5 cm distance from the tumour projecting towards the bladder neck.

For this pilot study, samples of the first three consecutively enrolled patients in the SILENT-EMPIRE study were analysed.

For sample preparation of the FFPE samples, the QIAGEN Supplementary Protocol [23] was used with the Qiagen Deparaffinization Solution (Qiagen, Venlo, the Netherlands, cat. no. 19093).

For the purification of the FF and FFPE samples after Deparaffinization, the ZymoBiomics DNA Mini Kit (ZymoResearch, Irvine, CA, USA) for DNA isolation was used according to the manufacturer’s instructions [24].

The concentration of isolated DNA was measured using PicoGreen measurement (Quant-iT™ PicoGreen™ dsDNA Assay Kit, ThermoFisher, Waltham, MA, USA). The integrity of a random sample was checked by agarose gel electrophoresis.

To prepare the library, sample quality control was conducted, and Nextera (Illumina, Zürich, Switzerland) two-step PCR amplification was performed using the primer set 515F × 806R (V3-V4 region of 16S rRNA) (Supplemental Table S1). After PCR product purification, quantification, and equimolar pooling, the paired-end sequencing (2 × 250 bp) of amplicon libraries was carried out on an Illumina MiSeq platform at Microsynth AG (Balgach, Switzerland).

QIIME2 was used to analyse the data. After checking the data quality, DADA2 was used for denoising to merge the paired reads and generate amplicon sequence variants (ASVs) to ensure sufficient depth for capturing most features [25]. An optimal sampling depth was selected by performing alpha rarefaction analysis to evaluate alpha and beta diversity measures, resulting in a setting cut-off of 19,000 reads per sample. Taxonomy was assigned to ASVs using the classify–learn Naïve Bayes classifier against the pre-trained Naïve Bayes silva-138-99-nb-classifier trained against Silva (release 138) full-length sequences [26].

### 2.2. Patient Data Acquisition

Patient data were collected by accessing the clinical information system of the University Hospital of Zurich, which was stored in the electronic data capture software REDCap database (Research Electronic Data Capture, Vanderbilt University, Nashville, TN, USA, v 9.3.8).

### 2.3. Statistics

For microbiome analysis (16S rRNA sequencing), the difference in alpha diversity (diversity of species within each sample) and beta diversity (diversity of species between samples) between FF and FFPE samples and between cancerous and non-cancerous tissue was assessed using the Shannon, inverse Simpson, and Gini–Simpson indices and the Pielou evenness index. Significance was determined using the Wilcoxon test. Statistical analysis was performed using R (R-Foundation, Vienna, Austria, v 4.1.0).

### 2.4. Ethics

The study has been approved by the Cantonal Ethics Committee Zurich (2021-01783, date: 12.10.2021), and it is being conducted in accordance with the Declaration of Helsinki and Good Clinical Practice.

## 3. Results

### 3.1. Patient Characteristics

Samples of the first three consecutively enrolled patients in the SILENT-EMPIRE study were included. Eleven samples were available for the final analysis: three samples with non-cancerous tissue archived in FF and FFPE and three samples of cancerous tissue archived in FFPE. Additionally, two FF tumour samples were available as, in one case, the collected amount of resected tissue did not allow for obtaining both an FF and FFPE sample, so only an FFPE tumour sample was collected. All three patients analysed in this study were male and were 68, 71, and 78 years old. Histopathology revealed a pTa tumour in all three patients, with one classifying as low grade and two as high grade. All patients had a history of smoking. No antibiotic treatment within one month before sample collection was recorded in any of the patients’ histories (Table 1).

### 3.2. DNA-Extraction

DNA extraction via PCR confirmed good data quality. It became empirically apparent that the analysed tissue samples had to have a minimum size of 25 mm^2^ (5 mm × 5 mm) to obtain satisfactory DNA extraction results. One tumour sample resulted in less than 19,000 reads.

### 3.3. Alpha Diversity

**Figure 1:**
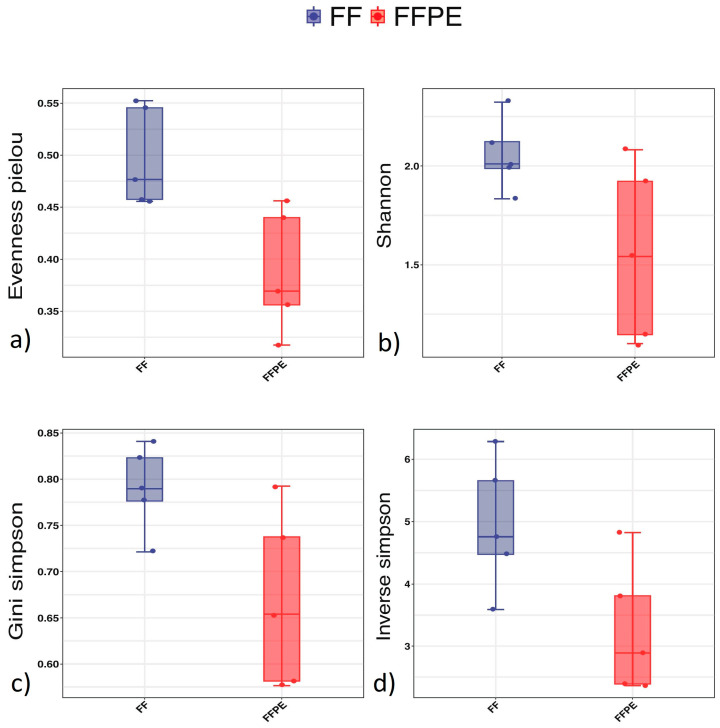
Alpha diversity (FF ^1^ vs. FFPE ^2^). ^1^ FF = fresh frozen; ^2^ FFPE = formalin-fixed paraffin-embedded. Alpha diversity: (**a**) shows a significantly higher evenness Pielou in FF compared to FFPE samples (*p* = 0.016); the Shannon index (**b**) (*p* = 0.095), Gini–Simpson index (**c**) (*p* = 0.095), and inverse Simpson index (**d**) (*p* = 0.095) were not significant.

When comparing alpha diversity in FF and FFPE samples, the evenness Pielou showed significantly higher diversity in FF tissue (*p* = 0.016). When examining the Shannon, inverse Simpson and Gini–Simpson indexes, a trend towards higher alpha diversity in FF was visible without reaching statistical significance (Shannon *p* = 0.095, inverse Simpson *p* = 0.095, Gini–Simpson *p* = 0.095) (Figure 1).

The core abundance index was significantly higher in the FFPE group compared to FF (*p* = 0.008).

**Figure 2:**
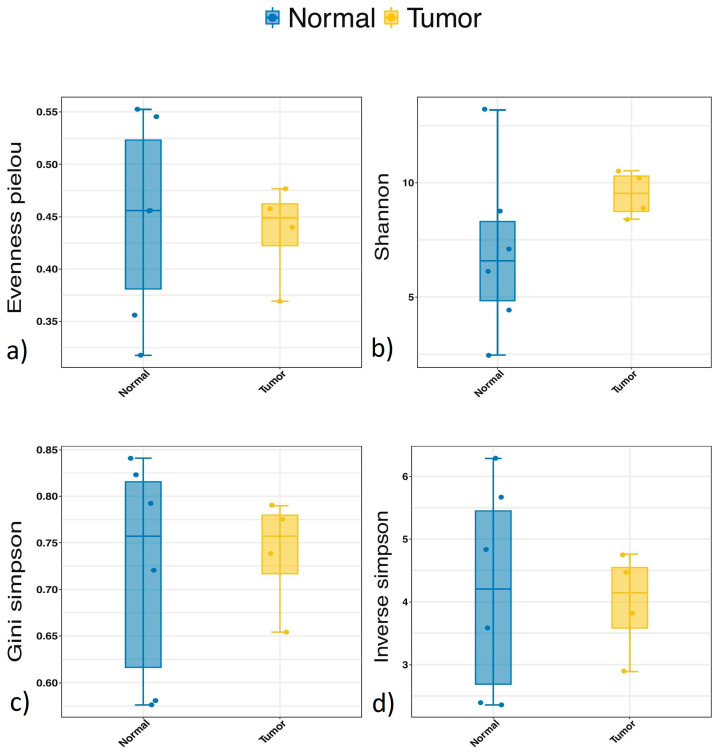
Alpha diversity (tumour vs. non-tumour). No significant differences in alpha diversity were detected between normal (non-cancerous) and tumour (cancerous) tissue in the same bladder cancer patients analysed by evenness_Pielou (**a**) (*p* = 1); Shannon index (**b**) (*p* = 0.914); Gini–Simpson index (**c**) (*p* = 0.914); and inverse Simpson index (**d**) (*p* = 0.914).

When comparing alpha diversity between cancerous and non-cancerous tissue, no significant differences in diversity, evenness, or core abundance could be detected (Figure 2).

### 3.4. Beta Diversity

**Figure 3:**
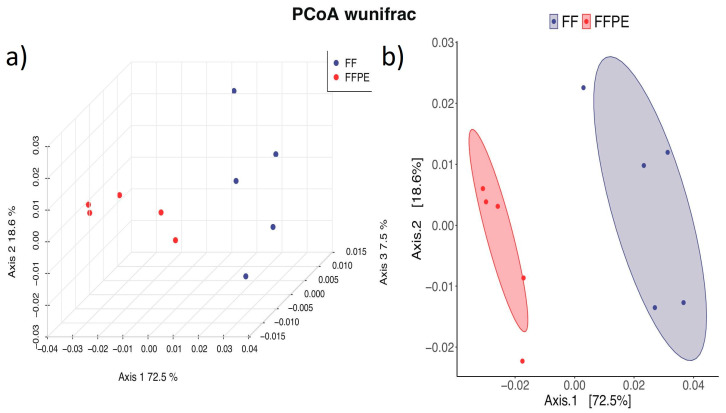
Beta diversity (FF ^1^ vs. FFPE ^2^). ^1^ FF = fresh frozen; ^2^ FFPE = formalin-fixed paraffin-embedded. Beta diversity: the principal coordinate analysis (PCoA) plot shows a clear separation between the FF and FFPE groups based on weighted UniFrac distances (**a**). The larger confidence ellipse area indicates a higher intragroup variability in FF compared to FFPE (**b**).

The principal coordinate analysis (PCoA) plot revealed a clear separation between the FF and FFPE groups based on weighted UniFrac distances (Figure 3). The centroids of the confidence ellipses for each group are distinctly separated along Axis.1, which accounts for 72.5% of the variation, demonstrating that the preservation method significantly impacts microbial community composition. Also, the FF samples showed higher intragroup variability than the FFPE, indicated by a larger confidence ellipse area.

### 3.5. Taxonomy

*Proteobacteria* were most commonly identified at the Phylum level, followed by *Actinobacteriota*. No significant differences between different storage methods (FF vs. FFPE) or tissue types (cancerous vs. non-cancerous) could be detected (Supplemental Figure S1).

At the family level, *Burkholderiaceae* were the most prevalent in all tissue and storage types. Other commonly identified families included *Comamonadaceae, Micrococcaceae, Xanthobacteraceae, Rhizobiaceae*, and *Sphingomonadaceae* (Supplemental Figure S2). *Rhiziobiales* (*p* = 0.038), *Rhizobiaceae* (*p* = 0.038), and *Nocardiaceae* (*p* = 0.020) were significantly more prevalent in FF tissue compared to FFPE samples.

Burkholderia-Caballeronia-Paraburkholderia was most abundant at the genus level. Other abundantly identified genera included Aquabacterium, Bradyrhizobium, Mesorhizobium, Sphingomonas, and Ralstonia (Supplemental Figure S3). Bradyrhizobium (*p* = 0.038), Mesorhizobium (*p* = 0.020), and Rhodococcus (*p* = 0.020) were significantly increased in FF-processed samples.

No significant difference between cancerous or non-cancerous tissue could be detected at the family or genus level.

## 4. Discussion

Our research demonstrates the viability of analysing the bladder microbiome through FF and FFPE tissue samples, proving that FFPE samples can be used in our main study. This is particularly relevant, as most histological analysis in clinical practice utilizes FFPE tissue due to its more efficient and cost-effective production process [18,20,27]. As a result, any findings based on FFPE samples have a higher likelihood of being translated and implemented into clinical practice, potentially impacting clinical guidelines. Additionally, our findings emphasize the importance of exploring different tissue types in microbiome studies depending on the scope of the research question (clinical focus vs. basic science) [18,19,20].

This is the first study directly comparing the microbiome biodiversity of bladder tumour tissue simultaneously in FF and FFPE samples. We demonstrated that microbiome alpha diversity measured by the Pielou evenness index decreased in FFPE compared to FF. Also, a lower core abundance index was found in FFPE, indicating that the core microbiome comprises fewer individual species of bacteria. Both findings suggest that a loss of information might occur during the FFPE preparation process.

This finding is supported by previous research. Schoffmann et al. [21] analysed proteome composition in FF and FFPE samples and found a higher degree of protein modification in the FFPE group. An analysis of extracted RNA from FFPE by Bøttcher et al. [28] showed a higher degree of RNA degradation when compared to fresh or frozen tissue. Oh et al. [29] compared the accuracy of whole genome sequencing in FFPE and FF samples and observed more artefacts in the FFPE stored samples. Notably, the studies mentioned above concluded that RNA analysis and whole genome sequencing were still possible in the FFPE group despite the loss of information because the extracted genetic data from FFPE are still sufficient [28,29]. This supports our conclusion that microbiome analysis is feasible in FFPE samples and, despite encountering a certain amount of information loss, still provides sufficient data to provide new insights into the microbiome under the premise of an adequate sample size calculation.

Several studies have shown differences between the microbiome found in patients with bladder cancer and compared to healthy individuals. Pederzoli et al. [30] identified the genus *Burkholderia* as more abundant in tumour tissue than in the normal bladder tissue of healthy controls. Also, comparing the microbiome extracted from cancer tissue compared to that from the urothelium of cancer-free patients, Mansour et al. [31] found *Staphylococcus, Corynebacterium*, and *Oxyphotobacteria* to be more common in cancer tissue. So far, no statements on the causality can be made. Further research is needed to determine whether the differences in the microbiome between healthy individuals and cancer patients are caused by cancer-induced processes or whether the microbiome changes facilitate cancer development.

When comparing alpha diversity in the cancerous and non-cancerous tissue of BC patients, we could not detect a significant difference. If confirmed in future research, this could indicate a possible field effect of microbiome alteration in tumour patients. Regarding the scarce literature, other study groups arrived at contradicting conclusions. The study by Liu et al. [32] compared the microbiome diversity of cancer tissue to the non-cancerous tissue of cancer patients. The DNA extraction was performed directly after sample collection without prior freezing or formalin fixation and showed a significant difference in the Shannon index.

Examining the FF samples of cancerous and non-cancerous tissue in cancer patients, Parra-Grande et al. [33] showed a decreased alpha diversity in cancer tissue. Additionally, they found *Actinobacteria* more prevalently in non-cancerous tissue.

Both studies mentioned above examined at least partially MIBC samples, making our small study the first focusing solely on NMIBC. It remains unclear if a simultaneous change in tumour microbiome composition occurs during cancer progression. The existing data do not appear conclusive, calling for additional data, preferably with a larger sample size, which we will provide in our main study.

While many studies have examined the urinary microbiome of BC patients via the analysis of urine samples [13,34,35,36], less is known about the microbiome found within the tumour tissue itself. Focusing on the microbiome in the urine has several shortcomings. Studies have shown, for example, a difference between the microbiome of the bladder and the urethra, which could lead to contamination during sample collection [37].

Considering the types of bacteria identified, the most abundant phylum in our study was *Proteobacteria*, followed by *Actinobacteriota*. Supporting our findings, Liu et al. [32] identified *Proteobacteria* as the most common phylum in BC tissue, followed by *Firmicutes, Bacteroides*, and *Actinobacteria*. Similarly, Mansour et al. [31] found *Firmicutes*, *Actinobacteria, Proteobacteria*, *Bacteroidetes*, and *Cyanobacteria* to be most prevalent at the genus level in BC tissue.

Analysing the cancerous tissue and non-cancerous tissue of cancer patients, Parra-Grande et al. [33] concluded that the genera *Firmicutes*, *Bacteroidetes*, *Proteobacteria*, and *Actinobacteria* were most abundant, supporting our results as well.

At the genus level, *Burkholderia-Caballeronia-Paraburkholderia*, *Aquabacterium*, *Bradyrhizobium*, *Mesorhizobium*, and *Sphinogomonas* were commonly identified in our samples. Liu et al. [32] reported finding high quantities of *Cupriavidus*, *Sphinomonas*, *Brucellae*, and *Actinetobacter,* thus partway confirming our findings. Mansour et al. [31], on the other hand, found *Bacteroides*, *Akkermansia*, *Klebsiella*, and *Clostridium sensu stricto* to be the most common genera.

Again, the existing data do not provide conclusive results. So far, only a few studies have covered various sampling and tumour types of different populations and nationalities. Considering the possible impact of these different phyla and genera on cancer progression, more reliable data are urgently needed.

The main limitation of the current study is the small sample size. It should be noted, however, that this study serves as a preliminary investigation, and therefore the sample size is not of paramount significance. Further, it was not possible to control for factors such as age, gender, and ethnicity, which may have influenced the microbial composition. Despite the limited number of samples in this pilot study, we demonstrated the feasibility of conducting microbiome analysis on formalin-fixed paraffin-embedded (FFPE) and fresh frozen (FF) samples.

## 5. Conclusions

Our study demonstrated that microbiome analysis is feasible in FF- and FFPE-processed bladder tissue samples. This could simplify further research concerning the bladder microbiome using readily available FFPE samples and aid in the translation of research findings into clinical practice.

## Figures and Tables

**Table 1:** Patient characteristics.

Patient	Age	Stage	Grade	Prior AB ^1^	Smoke Status	Primary/Recurrence
1	71	pTa	Low grade	None	ca. 10PY ^2^	Recurrence
2	68	pTa	High grade	None	ca. 20PY ^2^	Primary
3	78	pTa	High grade	None	ca. 40PY ^2^	Primary

^1^ AB = antibiotics; ^2^ PY = pack years.

## Data Availability

The datasets generated and analysed during the current study are available in the Zenodo repository: Bieri, U., Enderlin, D., Gadient, J., Surber, J., Eberli, D., & Poyet, C. (2024). Amplicon Sequencing Analysis Dataset/Towards Reliable Methodology: Microbiome Analysis of Fresh Frozen vs. Formalin-Fixated-Paraffin-Embedded Bladder Tissue Samples: A Feasibility Study (1.0). Zenodo. https://doi.org/10.5281/zenodo.11010303. The dataset includes raw sequencing data, quality assessment reports, analysis results, and downstream visualizations. It covers OTU summaries, taxonomic aggregations, and sample diversity measures. The data are provided in various formats, including TSV, FASTA, HTML, PDF, XLSX, and R data objects. The dataset is freely accessible under the Creative Commons Attribution 4.0 International (CC BY 4.0) license. All data needed to evaluate the conclusions in the paper are present in the dataset.

## Supplementary Materials

The following supporting information can be downloaded at: https://www.mdpi.com/article/10.3390/microorganisms12122594/s1, Table S1: Tbl. 1, Figure S1: Taxonomy Phylum, Figure S2: Taxonomy Family, Figure S3: Taxonomy Genus. Reference [38] is cited in the supplementary materials.
