# Supplementary material for: Towards Reliable Methodology: Microbiome Analysis of Fresh Frozen vs. Formalin-Fixed Paraffin-Embedded Bladder Tissue Samples: A Feasibility Study
Source: Microorganisms. 2024 Dec 15;12(12):2594. doi: 10.3390/microorganisms12122594 (PMC11677477; doi:10.3390/microorganisms12122594)
Supplement: Supplementary file 1 [file microorganisms-12-02594-s001.zip › microorganisms-3345303-supplementary.pdf]

Supplemental

Table S1 Primer Sequence

Primer Name	Sequence (5′-3′)	Region	Size (bp)	Source
341F	CCTACGGGNGGCWGCAG	V34	~460	[2]
805R	GACTACHVGGGTATCTAATCC			

[2] Klindworth et al. (2013) Evaluation of general 16S ribosomal RNA gene PCR primers for classical and next-generation sequencing-based diversity studies, Nucleic Acid Res, 41, e1.

Figure S1 Taxonomy Phylum

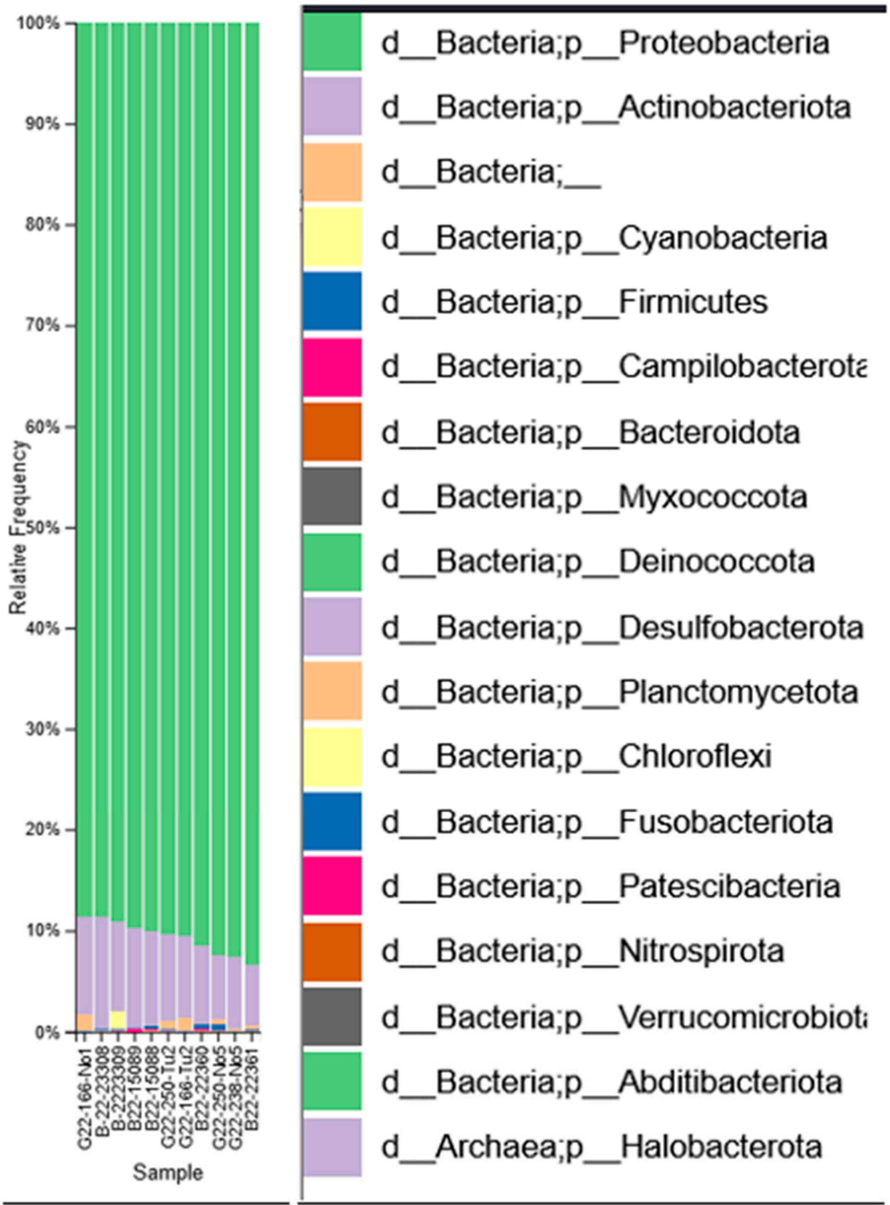

Depicting the most commonly identified phyla per analysed sample. If Information is missing, sequencing information was not sufficient for phyla allocation.

Figure S2 Taxonomy Family

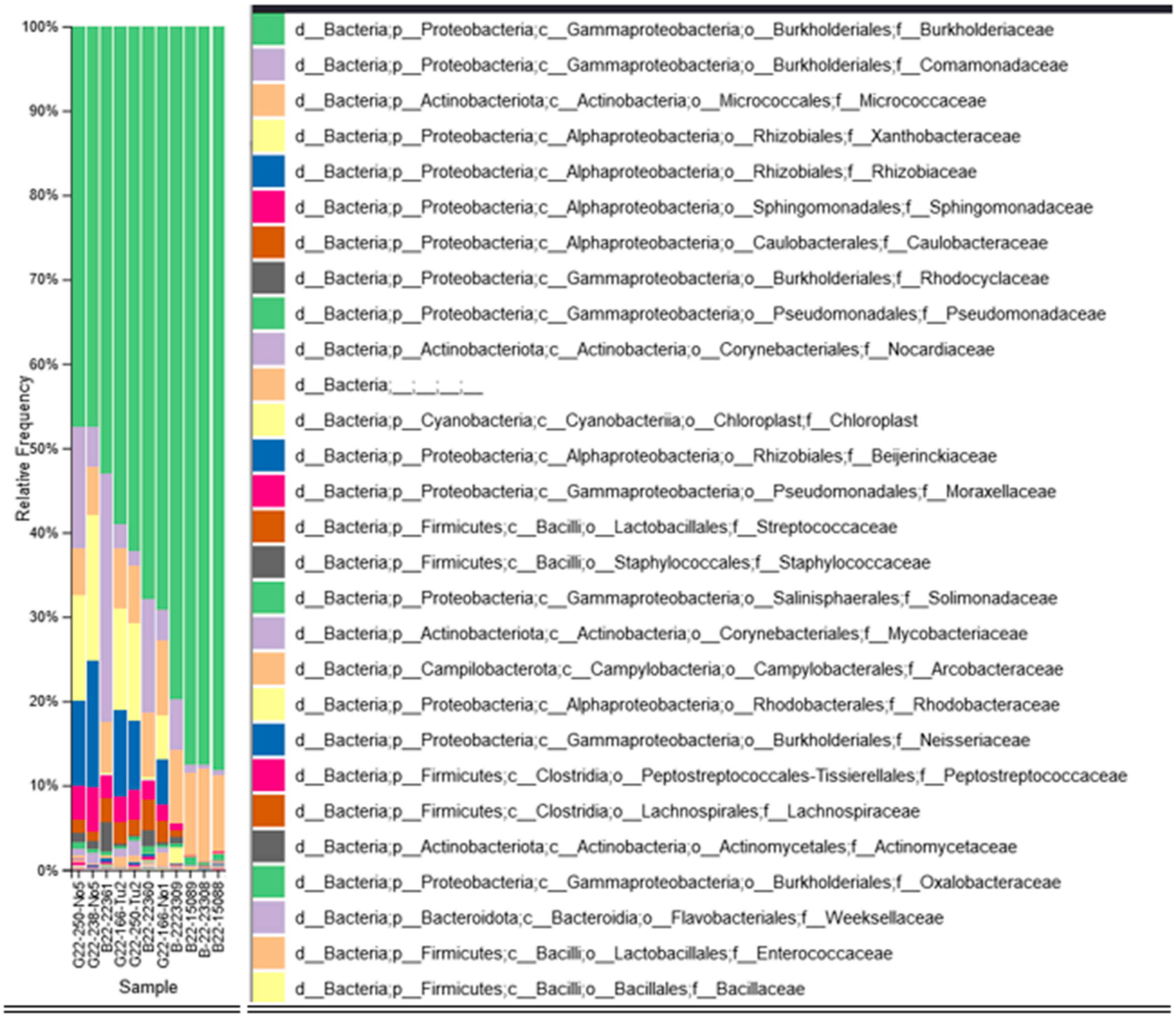

Depicting the most commonly identified family per analysed sample. If Information is missing, sequencing information was insufficient for phyla/family allocation.

Figure S3 Taxonomy Genus

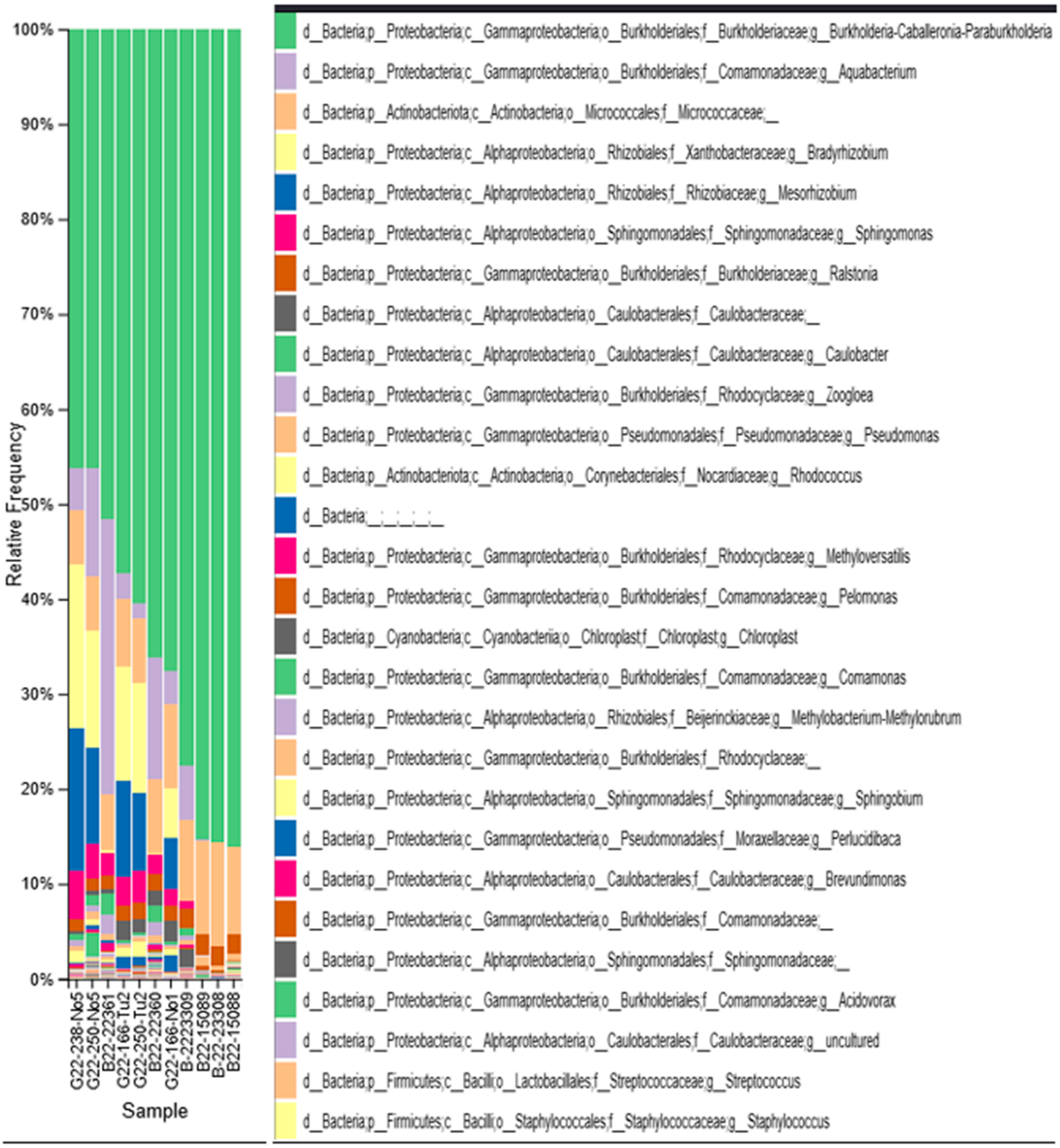

Depicting the most commonly identified geni per analysed sample. If Information is missing, sequencing information was insufficient for phyla/family/geni allocation.
